# A Physics-Based DNI Model Assessing All-Sky Circumsolar Radiation

**DOI:** 10.1016/j.isci.2020.100893

**Published:** 2020-02-08

**Authors:** Yu Xie, Manajit Sengupta, Yangang Liu, Hai Long, Qilong Min, Weijia Liu, Aron Habte

**Affiliations:** 1 Power Systems Engineering Center, National Renewable Energy Laboratory, Golden, CO 80401, USA; 2 Environmental and Climate Sciences Department, Brookhaven National Laboratory, 99 Rochester St., Upton, NY 11973, USA; 3 Computational Science Center, National Renewable Energy Laboratory, 15013 Denver West Parkway, Golden, CO 80401, USA; 4 Atmospheric Sciences Research Center, State University of New York, 251 Fuller Road, Albany, NY 12203, USA; 5 School of Atmospheric Physics, Nanjing University of Information Science and Technology, Nanjing, China

**Keywords:** Environmental Science, Energy Resources, Radiation Measurement

## Abstract

By investigating the long-term observations at Atmospheric Radiation Measurement (ARM) Southern Great Plains (SGP), we find that the routinely used Beer-Bouguer-Lambert law and the models that empirically separate direct normal irradiance (DNI) from measurements of global horizontal irradiance (GHI) have dramatic and unexpected bias in computing cloudy-sky DNI. This bias has led to tremendous uncertainty in estimating the electricity generation by solar energy conversion systems. To effectively reduce the bias, this study proposes a physical solution of all-sky DNI that computes solar radiation in the infinite-narrow beam along the sun direction and the scattered radiation falls within the circumsolar region. In sharp contrast with the other DNI models, this method uses a finite-surface integration algorithm that computes solar radiation in differential solid angles and efficiently infers its contribution to a surface perpendicular to the sun direction. The new model substantially reduces the uncertainty in DNI by a factor of 2–7.

## Highlights

• A dramatic and unexpected bias is found in the routine computation of DNI
• A physics-based model is developed to integrate circumsolar radiances
• DNI computation comprises scattered radiation in circumsolar region
• This new model leads to a remarkable improvement in assessing DNI

## Graphical Abstract

**Figure:**
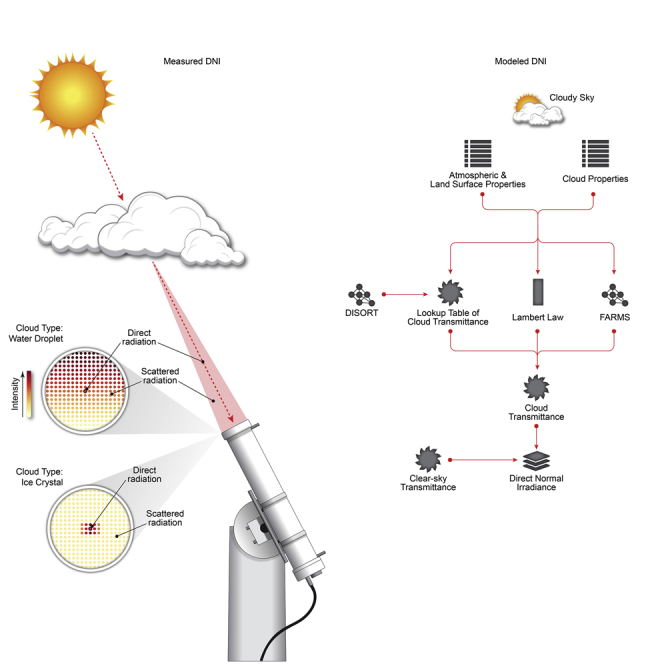

## Introduction

Direct normal irradiance (DNI) is one of the most used quantities to assess solar energy resource and is particularly crucial in evaluating or forecasting the performance of concentrating solar power systems (Desai et al., 2014, Lovegrove and Stein, 2012, Sengupta et al., 2018, Zhang et al., 2013). Radiative transfer models for atmospheric studies routinely assume that DNI is the solar radiation along a narrow beam straight from the sun that only covers the solar disk. Thus, the Beer-Bouguer-Lambert law, which formulates the transmittance of DNI in accordance with an exponential function of the mass extinction cross section and the solar path length in the atmosphere, has been consequently used in weather and climate research to numerically calculate direct solar radiation (Chandrasekhar, 1950, Kotchenova et al., 2006, Liou, 2002, Meador and Weaver, 1980, Mlawer et al., 1997, Stamnes et al., 1988, Wendisch and Yang, 2012, Xie and Liu, 2013, Xie et al., 2014).

DNI is often interpreted differently, however, in the study of solar energy or observation by surface-based pyrheliometers (Blanc et al., 2014, Raisanen and Lindfors, 2019). For instance, ISO 9488 (https://www.iso.org) defines direct irradiance by “the quotient of the radiant flux on a given plane receiver surface received from a small solid angle centered on the sun's disk to the area of that surface.” This “small solid angle,” also known as the circumsolar region, is recommended to be approximately 100 times larger than the average solar disk (Blanc et al., 2014, Raisanen and Lindfors, 2019, WMO, 2010). Thus, DNI is often associated with substantial amount of scattered solar radiation within the circumsolar region leading to distinct disagreements with the simulation/forecast based on the Beer-Bouguer-Lambert law. These disagreements have seriously affected the model performance in solar resource assessment and forecasting and test frameworks aimed at understanding the implementations of models.

Numerous solar energy models for computing clear-sky solar radiation take into account the scattered radiation in the circumsolar region by analytically or empirically modeling the Rayleigh scattering and the scattering by atmospheric aerosols (Badescu et al., 2012, Gueymard, 2003, Gueymard, 2012a, Ruiz-Arias and Gueymar, 2018). Although the typical clear-sky uncertainty in DNI simulation is noticeably greater than global horizontal irradiance (GHI) by a factor of 3–4 (Gueymard, 2012b), it is profoundly lower than cloudy-sky DNI owing to the strong forward scattering by clouds and the complexity in the computation of the radiative transfer within clouds (Sengupta et al., 2018, Sun and Liu, 2013). Most relevant research to date has focused on reducing the cloudy-sky uncertainties by developing regression functions to relate long-term measurements of GHI to DNI (Erbs et al., 1982, Gueymard and Ruiz-Arias, 2016, Yang and Boland, 2019). Similar to that, DNI can be also correlated with atmospheric properties, e.g., clearness index, using regression functions or artificial intelligence algorithms (Feng et al., 2018, Qin et al., 2018, Wang et al., 2016, Wang et al., 2019). Their applications, however, are often restricted by atmospheric and geographic circumstances used to determine the empirical coefficients and test the model performance.

Despite the capability to precisely compute solar radiation, radiative transfer models based on the numerical solution of the radiative transfer equation have been rarely used in solar energy research due to the excessive requirements on computing resources. To bridge the advantages of the solar energy models and radiative transfer models, Xie et al. (2016) proposed a Fast All-sky Radiation model for Solar applications (FARMS) that utilized the Rapid Radiative Transfer Model (RRTM) (Mlawer et al., 1997) to precompute and parameterize cloud transmittances for the possible cloud conditions and solar incident angles. The cloud parameterization was coupled with the clear-sky transmittance given by REST2 (Gueymard, 2008) to effectively compute GHI for all-sky conditions. The FARMS was enhanced to compute narrowband irradiances on tilted photovoltaic (PV) panels (referred to as FARMS-NIT) (Xie and Sengupta, 2018, Xie et al., 2018, Xie et al., 2019) where the cloud bidirectional reflectance distribution function (BTDF) was computed by a 32-stream DIScrete Ordinates Radiative Transfer (DISORT) model (Stamnes et al., 1988). The solar radiances were simultaneously computed in 2002 wavelength bands based on the cloud BTDF and the Simple Model of the Atmospheric Radiative Transfer of Sunshine (SMARTS) (Gueymard, 1995), and they were integrated over an inclined surface to estimate the spectral radiation received by a PV panel.

Following the previous studies, this work further extends the capability of FARMS for the computation of DNI (hereafter referred to as FARMS-DNI) that comprises the influence of the circumsolar radiation. In contrast to the decomposition models separating DNI from GHI observation, this new model is based on inputs from the atmospheric and land surface retrievals and a finite-surface integration algorithm that computes solar radiation in differential solid angles and efficiently infers its contribution to a surface perpendicular to the solar direction. FARMS-DNI can also serve as a decomposition model when GHI observation/simulation is available.

## Results

### The Decomposition of DNI

**Figure 1:**
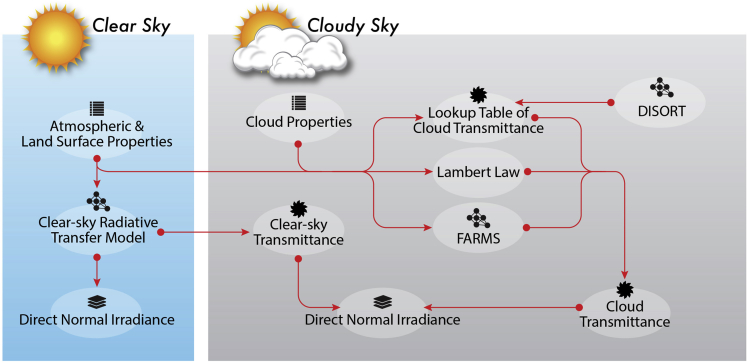
A Flowchart of the FARMS-DNI Model

**Figure 2:**
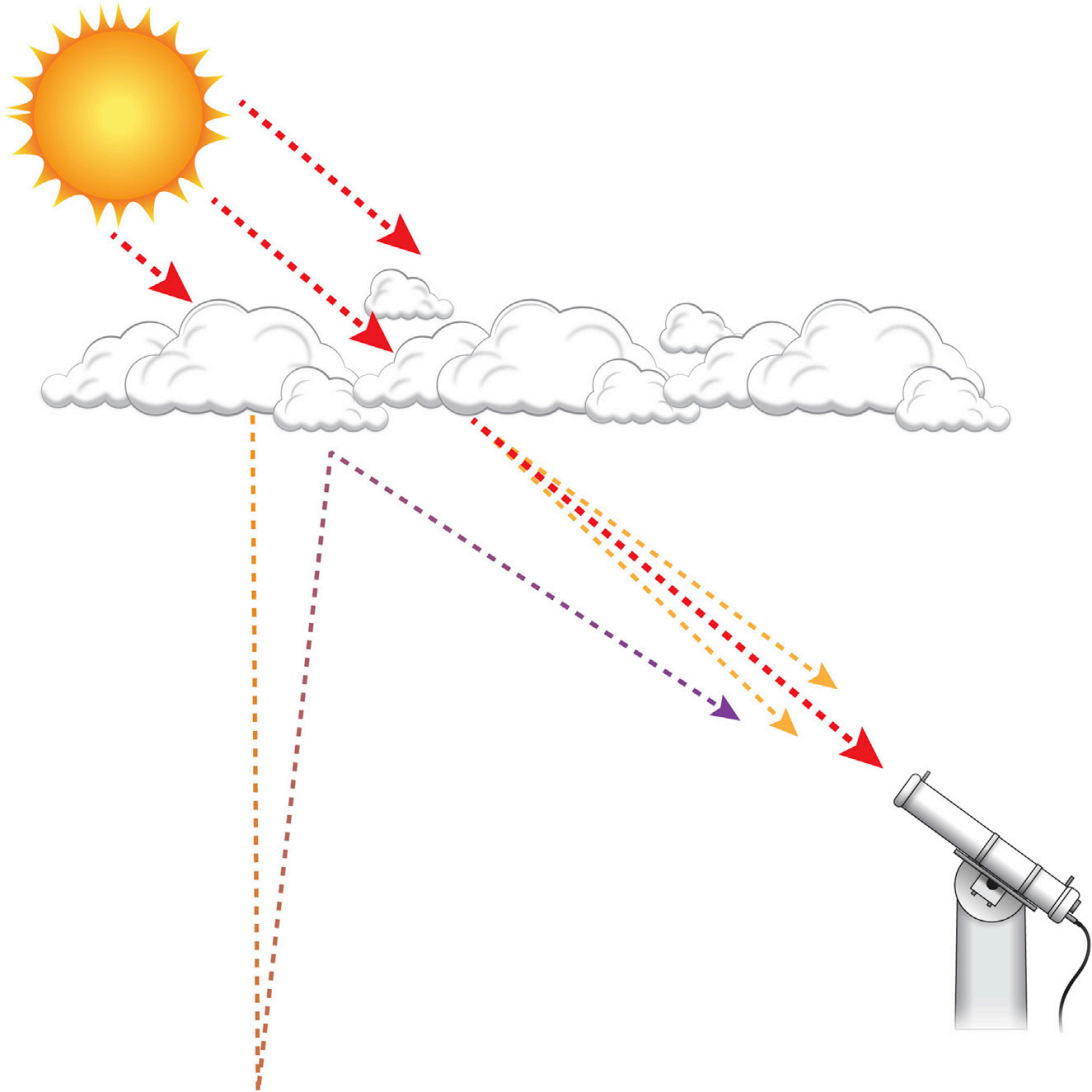
Illustrative Diagram of Cloudy-Sky DNI Observed by a Surface-Based Pyrheliometer The red, orange, and purple lines represent the transmission of the atmosphere in the infinite-narrow beam, the transmission related to the first-order diffuse radiation along the direct beam, and the transmission related to the multiple reflection along the direct beam, respectively.

Figure 1 summarizes the scheme of computing DNI by FARMS-DNI. For clear-sky conditions, the transmittance of direct radiation is affected by the atmospheric absorption, Rayleigh scattering, and the scattering by aerosols in the atmosphere, which can be computed by a clear-sky radiative transfer model, e.g., REST2 (Gueymard, 2008), and models developed by Bird and Hulstrom (1981) and Ineichen and Perez (2002), which is designed for simulating observations by a surface-based pyrheliometer. For cloudy-sky conditions, DNI is decomposed by three major components in the transmission through the atmosphere: (1) the transmission in the infinite-narrow beam, (2) the transmission related to the first-order scattered radiation in the circumsolar region, and (3) the transmission related to the multiple reflection in the circumsolar region (Figure 2).

Following the procedure of a number of radiative transfer models, the first term in DNI can be simply computed by the Beer-Bouguer-Lambert law (Liou, 2002) using the cloud optical thickness and the transmittance of the direct radiation in the clear atmosphere. Note that DNI is computed by considering only this first term in the conventional FARMS, which therefore should lead to an underestimation of DNI compared with surface-based observations. The transmission related to the first-order scattered radiation is computed in a diminutive solid angle corresponding to the circumsolar region where the clear-sky transmittance is given by REST2 (Gueymard, 2008) and the cloud transmittance is provided by a precomputed lookup table for possible cloud conditions and solar incident directions. According to the recommendation by the World Meteorological Organization (WMO) (2010), a 5° opening angle is assumed for the pyrheliometer on the land surface. The details in computing the cloud transmittance are specified in the next section. The transmission related to the multiple reflection in the circumsolar region is approximated by computing the downwelling irradiance by FARMS with an assumption of isotropic diffuse radiation in the multiple reflection between the cloud and land surface. The detailed procedure of computing the three components of DNI can be found in the Supplemental Information.

### A Finite-Surface Integration Algorithm and a Lookup Table for Inferring Cloud Transmittance

Following the previous studies (Xie and Sengupta, 2018, Xie et al., 2018, Xie et al., 2019), the transmittance related to the first-order scattered radiation, $T_{dd}^{cld}$, can be computed by a finite-surface integration algorithm for the circumsolar region:

(Equation 1) $$T_{dd}^{cld}=\frac{1}{\pi }\iint_{\Omega \left(\theta_{0}\right)}TF_{0t}^{cld}cos\theta sin\theta d\theta d\phi$$

where $\Omega \left(\theta_{0}\right)$ is the solid angle corresponding to the circumsolar region, $\theta_{0}$ is the solar zenith angle, θ is the zenith angle, ϕ is the azimuth angle, $TF_{0t}^{cld}$ is the cloud BTDF, and the subscripts “0” and “t” represent the solar incident and outgoing directions, respectively. In the direct solar beam, $TF_{0t}^{cld}$ is dependent on viewing azimuth angle but independent of the solar azimuth angle. The latter is thus assumed as 0°. $TF_{0t}^{cld}$ in the solar spectral region can be inferred from the computation of spectral radiances transmitted through the cloud:

(Equation 2) $$\begin{array}{c} TF_{0t}^{cld}=\frac{\pi \int_{\lambda_{1}}^{\lambda_{2}}I_{0t,\lambda }d\lambda }{\mu_{0}F_{0}} \end{array}$$

where $F_{0}$ is the extraterrestrial solar irradiance, $\mu_{0}$ is the cosine value of the solar zenith angle, $I_{0t,\;\lambda }$ is the spectral radiance transmitted through the cloud, λ denotes the wavelength, and $\lambda_{1}$ $\lambda_{2}$ represent the lower and upper limits of the wavelengths, respectively, in the solar spectral region.

To efficiently compute DNI, we utilize Equations 1 and 2 to develop a precomputed lookup table of cloud transmittance, i.e., $T_{dd}^{cld}$, for the possible cloud properties and solar and viewing directions within the circumsolar region. Following the development of FARMS-NIT (Xie and Sengupta, 2018, Xie et al., 2018, Xie et al., 2019), solar radiance transmitted through the cloud, i.e., $I_{0t,\;\lambda }$, is computed in 97 wavelengths from 0.28 to 4.0 μm. The broadband cloud BTDF, i.e., $TF_{0t}^{cld}$, is then given by the integration over the 97 wavelengths as given in Equation 2. As suggested by an analysis of the optimized number of terms in phase function expansion (Ding et al., 2009), the 64-stream DISORT model is implemented to compute the solar radiances for 43 solar zenith angles of 0°, 2°, …, 84°. For clouds composed of water droplets or ice crystals, 39 cloud optical thicknesses and 28 cloud effective particle sizes are selected over the ranges according to the observed cloud properties and previous modeling efforts (Minnis et al., 2011, Xie, 2010, Xie et al., 2012a, Xie et al., 2012b, Xie et al., 2016). The technical details of the wavelength spectrum in the computation, mixture schemes of cloud particles, cloud optical properties, and their ranging intervals are not reinstated here because they have been given by previous publications (Baum et al., 2011, Hu and Stamnes, 1993, Xie et al., 2011, Xie et al., 2019). In each solar zenith angle, approximately 200 differential solid angles are considered in Equation 2. The sets of zenith and azimuth angles are correspondingly determined and registered to the differential solid angles.

The high-performance computing (HPC) system, Peregrine, at the National Renewable Energy Laboratory (NREL) is intensively used in the development of the lookup table because of the tremendous computing cost required by the 64-stream DISORT with multiple dimensions in wavelength, cloud thermodynamic phase, cloud optical thickness and particle size, and solar and viewing geometries. Peregrine is a computing cluster platform comprising 58,752 Intel Xeon processor cores, including 6,912 E5-2670 SandyBridge, 24,192 E5-2695v2 IvyBridge, and 27,648 E5-2670v3 Haswell processor cores, providing a peak performance of 2.26 PetaFLOPS. The computation lasted for 3 months with a total consumption of approximately 400,000 node hours; approximately 7,200 processor cores were simultaneously utilized on average, and more than 14,000 processor cores were recruited in the off-peak hours.

**Figure 3:**
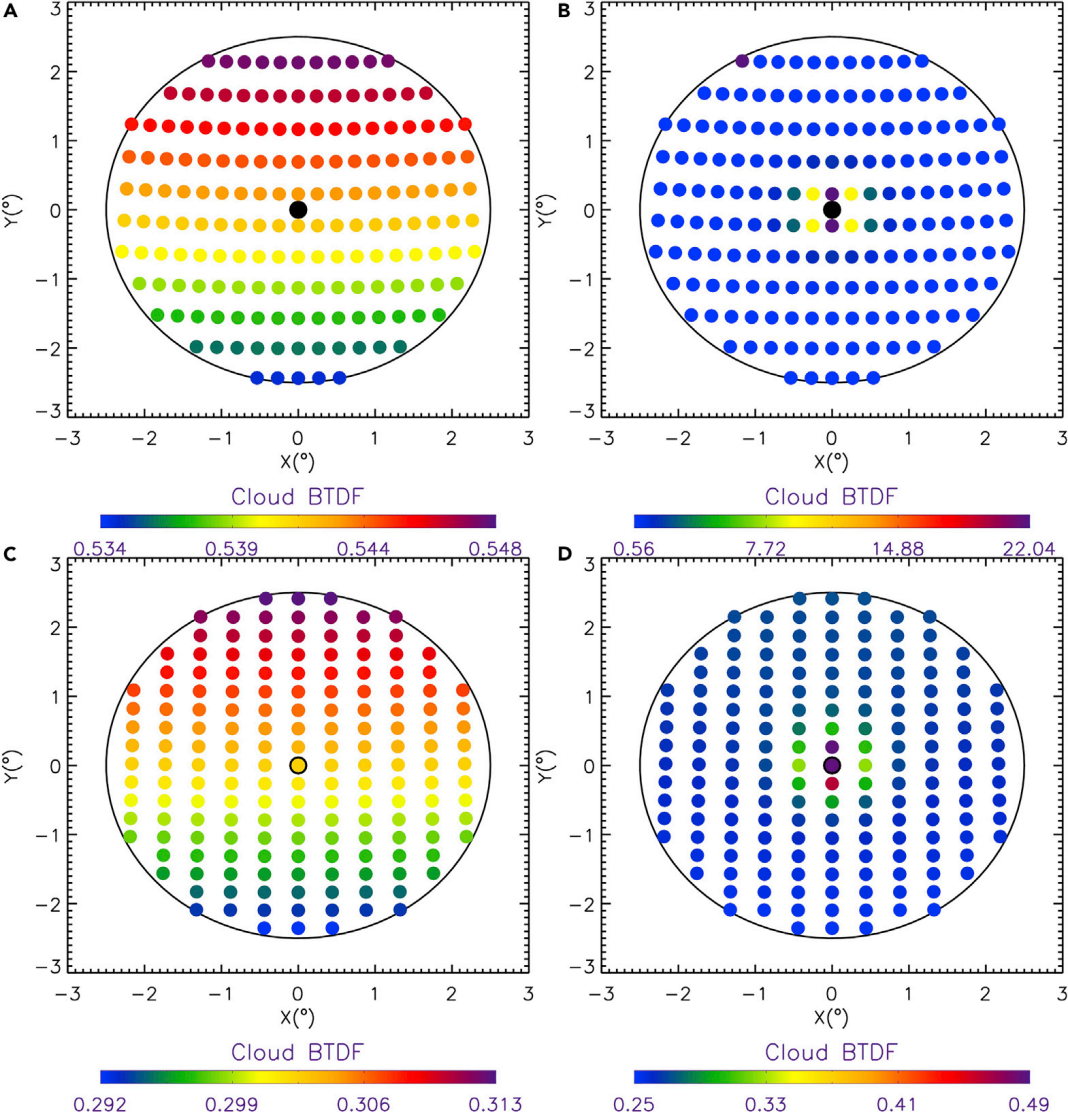
Cloud BTDF Cloud BTDFs of (A) water and (B) ice clouds on the cross section of the circumsolar region when the cloud optical thickness is 12 and the solar zenith angle is 30°. The cloud effective particle sizes are 10 and 100 μm for water and ice clouds, respectively. The black circle represents the cross section of the circumsolar region. (C) and (D) are the same as (A) and (B), respectively, except that the solar zenith angle is 60°.

Figure 3 demonstrates the cloud BTDF of water and ice clouds on the cross section of the circumsolar region when the cloud optical thickness is 12 and solar zenith angle is 30°. The round dots in Figure 3 represent the elements of solid angle for each differential μ and ϕ. The colors denote the magnitude of the cloud BTDF. For water clouds, slight variations in cloud BTDF are shown in all directions. For ice clouds, the transmittance of the solar radiation is much more significant around the direct beam, which is caused by the strong forward scattering by ice cloud particles. This is also shown in Figure 3, when the solar zenith angle is 60°. For both water and ice clouds, however, the magnitudes of the cloud BTDF are reduced because of the increased photon path within the cloud.

### Data for the Test, Improvement, and Evaluation of FARMS-DNI

The concept of the FARMS-DNI presented in the previous sections is based on idealized input data of the atmospheric and land surface properties. For future applications on solar resource assessment and forecasting, it is crucial to investigate the model performance with reliable observations, further calibrate the data and model according to the test, and validate it using the observations and state-of-the art models.

When instrumental observations are used as the model inputs and the references for model outputs, data selection and quality substantially affect the accuracy and credibility of the model evaluation. Because of that, we select data from two surface sites that enjoy a worldwide reputation for excellent and consistent data quality throughout decades: NREL's Solar Radiation Research Laboratory (SRRL) and the Atmospheric Radiation Measurement (ARM) Southern Great Plain (SGP) site. Although data from other surface sites, e.g., the Surface Radiation Budget Network (SURFRAD), which is maintained by the National Oceanic and Atmospheric Administration (NOAA), also deliver decent quality, the long-term data from the two selected sites should provide a good starting point for understanding the model performance.

Table 1 briefly describes the measurement sites and data used in this study. According to the geographic locations, the two sites belong to different climate zones with distinct elevations. For NREL's SRRL, the data quality is ensured by daily instrument maintenance with automated data quality control and internal consistency check using NREL's SERI-QC method. More details on the data quality and a comparison with the NOAA's SURFRAD has been reported by Anderberg and Sengupta (2014). We selected cloud fraction estimated by a Yankee total sky imager and surface pressure and albedo measured by a Vaisala pressure transmitter and an inverted pyranometer, respectively, both mounted 2 m above ground level. The GHI and DNI were measured by a CMP22 pyranometer and a CHP1 pyrheliometer mounted on an automatic sun tracker, respectively. The observations used in this study were from September 1, 2008 to December 1, 2017 with a temporal resolution of 1 min, including 336,972 cloudy-sky scenarios where the cloud fraction is greater than 0.95. The solar zenith angle was computed by the Solar Position Algorithm (SPA) (Reda and Andreas, 2004).

**Table 1:** Description of the Measurement Sites and Data Used in This Study

	NREL SRRL	ARM SGP
Latitude (°North)	39.742	36.605
Longitude (°West)	105.18	97.485
Elevation (meter)	1829	318
Time zone	UTC-6	UTC-5
Period	9/1/2008-12/1/2017	1/2/1998-12/31/2014
Resolution (minute)	1	15
Scenario	336,972	67,939

For ARM SGP, we used the decade-long measurements from January 2, 1998 to December 31, 2014 at the central facility at Lamont, Oklahoma. The GHI and DNI corresponding to 67,939 cloudy-sky scenarios were extracted from the Shortwave Flux Analysis (SWFA) value-added product (VAP) in a 15-min resolution. The direct and diffuse radiation data have passed an automated data quality check as introduced by Long and Gaustad (2004). The measurement and process of the SWFA VAP data were previously reported (Long and Ackerman, 2000, Long and Gaustad, 2004) and hence, they are not reiterated here.

In this study, the NREL's SRRL data are used in the initial test and improvement of FARMS-DNI. The ARM SGP data are used to evaluate the improved model.

### Cloud Optical Thickness Scaling for Improving the Computation of Forward Scattering

In DISORT, the single-scattering phase function of a cloud is approximated by an expansion of Legendre polynomials with a finite number of terms (Liou, 2002, Stamnes et al., 1988). To reduce the required expansion terms and thus minimize the overall computing time, we utilize the Delta-M method that truncates the forward peak in the single-scattering phase function of the cloud using a Dirac delta function (Wiscombe, 1977). Although the bias in the approximated phase function decreases when increasing the expansion terms, limited Legendre polynomials might not well estimate the phase function, especially in the directions around the forward scattering.

**Figure 4:**
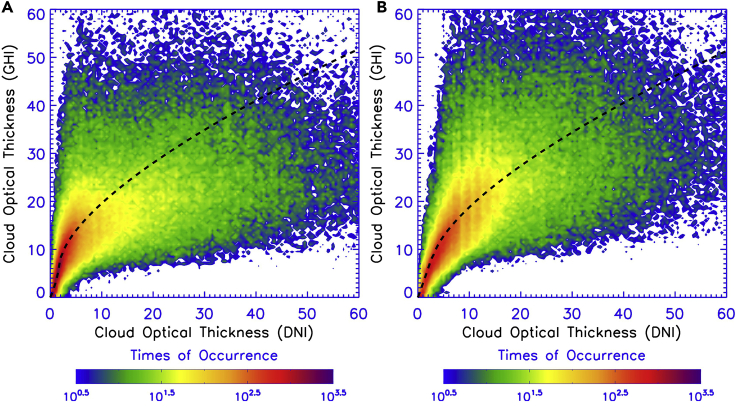
Comparison of Cloud Optical Thicknesses Retrieved Using the Observed GHI and DNI at NREL SRRL The retrievals are accomplished by matching the observation with the model simulation using (A) a water and (B) an ice cloud model. The dashed lines represent the relation between the retrievals from GHI and DNI.

Figure 4 illustrates cloud optical thicknesses retrieved using the surface-based observations of solar radiation at the NREL SRRL. The retrievals are accomplished by matching the GHI and DNI observation with the model simulation by FARMS and FARMS-DNI, respectively. For water and ice clouds, the cloud effective particle sizes are assumed to be 10 and 50 μm, respectively. For both clouds, the cloud optical thickness estimated by GHI is averagely larger than that estimated by DNI. Because GHI computed by FARMS has an excellent agreement with surface observations (Xie et al., 2016), this indicates an underestimation in the DNI simulation by FARMS-DNI, whereas a clear nonlinear relationship is visible between the cloud optical thickness determined by GHI and DNI.

According to the previous studies (Min and Duan, 2005, Min et al., 2004, Raisanen and Lindfors, 2019), the error in the computation of cloud forward scattering can be remedied by scaling the cloud optical thickness used in the radiative transfer model. The scaling function is derived by the Levenberg-Marquardt least-squares fit algorithm using the cloud retrieval at the NREL SRRL, as shown by the dashed lines in Figure 4. The resulting scaling functions and parameters are expressed as follows:

For a water cloud,

(Equation 3a) $$\begin{array}{c} \tau_{DNI}=\{\begin{array}{c} 0.254825\tau_{GHI}-0.00232717\tau_{GHI}^{2}+5.1932\times 10^{-6}\left[1+0.07\left(8-\tau_{GHI}\right)\right]\tau_{GHI}^{3}\;for\;\tau_{GHI}<8\\ 0.2{\left(\tau_{GHI}-8\right)}^{1.5}+2.10871\;for\;\tau_{GHI}\geq 8 \end{array} \end{array}$$

where $\tau_{DNI}$ and $\tau_{GHI}$ are the cloud optical thicknesses estimated by DNI and GHI, respectively.

For an ice cloud,

(Equation 3b) $$\begin{array}{c} \tau_{DNI}=\{\begin{array}{c} 0.345353\tau_{GHI}-0.00244671\tau_{GHI}^{2}+4.74263\times 10^{-6}\tau_{GHI}^{3}\;for\;\tau_{GHI}<8\\ 0.2{\left(\tau_{GHI}-8\right)}^{1.5}+2.91345\;for\;\tau_{GHI}\geq 8 \end{array} \end{array}$$

The consequence of Figure 4, in view of the accordance between surface-based GHI observation and that computed by FARMS and satellite-based retrieval of cloud optical thickness (Sengupta et al., 2018), is that satellite retrieval should be scaled by Equation 3 when used to compute DNI by FARMS-DNI. Clearly, as shown in Figure 4, FARMS-DNI also can numerically separate DNI from GHI observation when the latter is available and used to derive cloud optical thickness by FARMS and Equation 3, which is an obvious advantage compared with the other radiative transfer models.

## Discussion

### Validation

The computation of clear-sky DNI with REST2 is comparatively straightforward compared with the cloudy-sky models. In this stage, FARMS-DNI directly utilizes REST2 to compute clear-sky DNI, which has been evaluated with a few dozens of models in the same domain (Badescu et al., 2012). This study further investigates the performance of cloudy-sky FARMS-DNI against the Beer-Bouguer-Lambert law and the empirical Direct Insolation Simulation Code (DISC) (Maxwell, 1987). For the Beer-Bouguer-Lambert law, the clear-sky transmittance is computed by the REST2, whereas the cloud optical thickness is retrieved using FARMS and GHI observation at the ARM SGP. Note that the cloud optical thickness retrieved by FARMS and GHI is used to represent observations or satellite-based retrievals because previous studies show that GHI computed by satellite-determined cloud optical thickness and FARMS has a good agreement with surface observations (Sengupta et al., 2018). The retrieval of cloud optical thickness offers input for a fair comparison between the models. In the DISC model, DNI is empirically separated from GHI observation or simulation. In this study, cloudy-sky GHI observed at the ARM SGP is used as the input of the DISC model when the cloud fraction is greater than 0.95. For FARMS-DNI, the retrieved cloud optical thickness from the GHI observation is scaled by Equation 3 and then used to check the cloud transmittance from the lookup table that has been specified in the previous section.

**Figure 5:**
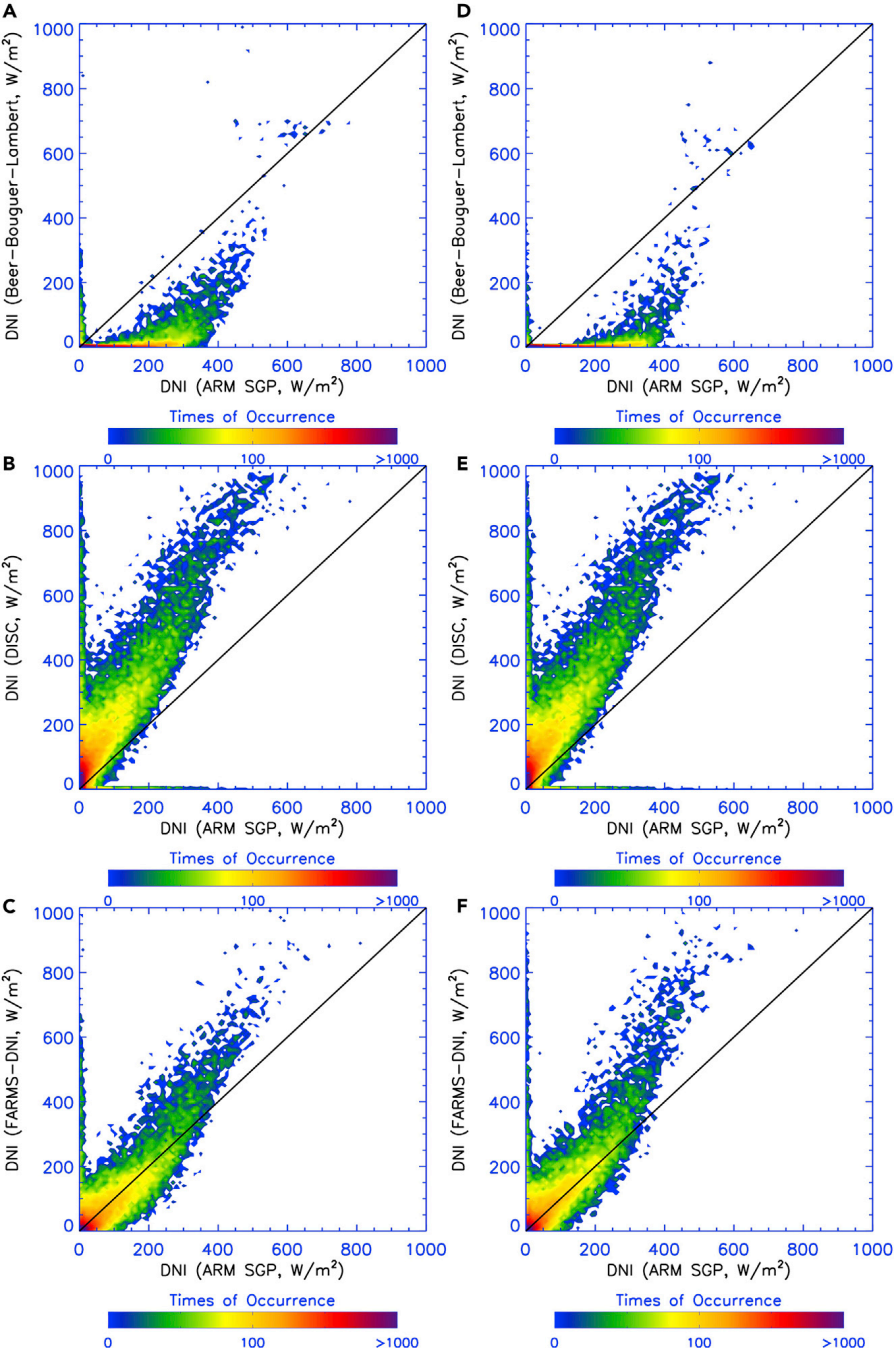
Comparison of the Observed and Computed DNIs Comparison of the DNI observed at the ARM SGP site with those computed by (A and D) the Beer-Bouguer-Lambert law, (B and E) DISC model, and (C and F) FARMS-DNI. A (A–C) water cloud model and an (D–F) ice cloud model are assumed in the retrieval of cloud optical thickness and the computation of DNI.

Figure 5 compares the DNI observed at the ARM SGP site with those computed by the Beer-Bouguer-Lambert law, DISC model, and FARMS-DNI where a water cloud model and an ice cloud model are assumed in the cloud retrieval and the computation of DNI. Note that Figure 5E duplicates the illustration in Figure 5B because the DISC model does not require cloud properties for the input variables. As shown in Figures 5A and 5D, DNI is significantly underestimated by the Beer-Bouguer-Lambert law owing to the neglect of the scattered energy in the circumsolar region. On the other hand, the DISC model substantially overestimates DNI, which is probably attributable to the empirical functions that are routinely determined under (partially) clear-sky dominated conditions. The inconsistent performance in the clear-sky and cloudy-sky conditions should thus be found in most decomposition models that use only one set of functions empirically determined by observations of all scenarios. Compared with the Beer-Bouguer-Lambert law and DISC model, FARMS-DNI has a much better agreement with surface-based observations irrespective of the cloud models used in the computation.

To quantitively understand the uncertainty of the three models, we investigate their mean bias error (MBE), mean absolute error (MAE), percentage error (PE), and absolute percentage error (APE), which are defined as follows:

(Equation 4a) $$\begin{array}{c} MBE=\frac{1}{n}\sum_{i=1}^{n}\left(DNI_{M}-DNI_{O}\right) \end{array}$$

(Equation 4b) $$\begin{array}{c} MAE=\frac{1}{n}\sum_{i=1}^{n}\left|DNI_{M}-DNI_{O}\right| \end{array}$$

(Equation 4c) $$\begin{array}{c} PE=\frac{\sum_{i=1}^{n}\left(DNI_{M}-DNI_{O}\right)}{\sum_{i=1}^{n}DNI_{O}}\times 100\% \end{array}$$

(Equation 4d) $$\begin{array}{c} APE=\frac{\sum_{i=1}^{n}\left|DNI_{M}-DNI_{O}\right|}{\sum_{i=1}^{n}DNI_{O}}\times 100\% \end{array}$$

where n is the number of the cloudy-sky scenarios at the ARM SGP and the subscripts “M” and “O” denote the model simulation and surface-based observation, respectively. In view of the statistics, the water cloud model has slightly better performance in the Beer-Bouguer-Lambert law and FARMS-DNI as compared with the ice cloud model (Table 2). This provides clear evidence that the water cloud model should be assumed in the cloud retrieval and the computation of DNI when FARMS-DNI serves as a decomposition model. The ice cloud model, however, should be employed when ice clouds are identified by surface or satellite-based remote sensing techniques.

**Table 2:** MBE, MAE, PE, and APE of the Computed DNI at the ARM SGP Using the Beer-Bouguer-Lambert Law, DISC, and FARMS-DNI

	MBE (Wm^−2^)	MAE (Wm^−2^)	PE (%)	APE (%)
Water cloud				
Beer-Bouguer-Lambert	−20.08	23.18	−74.98	86.56
DISC	41.21	44.86	153.85	167.47
FARMS-DNI	6.14	14.97	22.93	55.89
Ice cloud				
Beer-Bouguer-Lambert	−21.12	24.38	−78.85	91.01
DISC	41.21	44.86	153.85	167.47
FARMS-DNI	11.82	17.97	44.15	67.11

### Conclusions

By extending the idea behind FARMS and FARMS-NIT, a new physics-based model to compute all-sky DNI, which we have dubbed the FARMS-DNI, is created. In contrast to the Beer-Bouguer-Lambert law, FARMS-DNI computes DNI in the light of an overall effect of the solar radiation in the infinite-narrow beam along the solar direction and the scattered radiation falls within the circumsolar region. Compared with decomposition models, FARMS-DNI numerically computes solar radiation in differential solid angles within the circumsolar region and uses a finite-surface integration algorithm to infer their contribution to DNI. Although FARMS-DNI is based on the solution of the radiative transfer equation, it restores precomputed cloud transmittance, leading to significantly enhanced efficiency in the state-of-the-art atmospheric radiative transfer models. Comparison with the Beer-Bouguer-Lambert law and DISC model reveals FARMS-DNI renders remarkable improvement in DNI computation under cloudy-sky conditions.

### Limitations of the Study

In this study, cloud transmittances corresponding to DNI are precomputed by the 64-stream DISORT and provided by a lookup table in dimensions of cloud thermodynamic phase, cloud optical thickness, cloud effective particle size, and solar zenith angle. Despite the precision archived by the lookup table, a parameterization with plain functions is clearly a more favorable solution to provide cloud transmittance; this is particularly evident when the model becomes a component of more comprehensive models in solar forecasting or electric grid integration (Haupt et al., 2018, Xie et al., 2016). Nevertheless, the scaling functions of cloud optical thickness are determined using long-term data at the NREL SRRL. Although they have been validated using data at the ARM SGP, more comprehensive investigation is required to understand their applicability to extensive atmospheric and geographic conditions. More comprehensive comparisons with the other DNI models are also underway. Satellite products are required to further evaluate the performance of FARMS-DNI when it is used to compute DNI from the atmospheric and land surface properties. These subject matters have been left untouched by this paper but deserve future investigation.

## Methods

All methods can be found in the accompanying Transparent Methods supplemental file.

## Supplemental Information

Document S1. Transparent Methods and Figure S1
