# Supplementary material for: A Physics-Based DNI Model Assessing All-Sky Circumsolar Radiation
Source: iScience. 2020 Feb 8;23(3):100893. doi: 10.1016/j.isci.2020.100893 (PMC7038471; doi:10.1016/j.isci.2020.100893)
Supplement: Document S1. Transparent Methods and Figure S1 [file mmc1.pdf]

iScience, Volume *23*

Supplemental Information

A Physics-Based DNI Model

Assessing All-Sky Circumsolar Radiation

Yu Xie, Manajit Sengupta, Yangang Liu, Hai Long, Qilong Min, Weijia Liu, and Aron Habte

Supplemental Information

Transparent Methods

For cloudy-sky conditions, DNI is computed by considering three major components in the transmission through the atmosphere:

$$DNI = F_0(T_{d0} + T_{d1} + T_{d2}) \quad (S1)$$

where F_0 is the extraterrestrial solar irradiance, T_{d0} is the transmittance of the atmosphere in the infinite-narrow beam, T_{d1} is the transmittance related to the first-order scattered radiation in the circumsolar region, and T_{d2} is the transmittance related to the multiple reflection in the circumsolar region. The three components of the transmission in the direct radiation are illustrated in Fig. 2, which assumes a plane-parallel cloud in the atmosphere.

According to the Beer-Bouguer-Lambert law, T_{d0} can be given by

$$T_{d0} = T_{dd}^{clr} exp\left(-\frac{\tau}{\mu_0}\right) \quad (S2)$$

where μ_0 is the cosine value of the solar zenith angle and τ is the cloud optical thickness. T_{dd}^{clr} is the transmittance of the direct radiation in the clear atmosphere that can be computed by a clear-sky radiative transfer model. Following FARMS, the REST2 is used in this study. Note that DNI is computed by considering only T_{d0} in the conventional FARMS.

T_{d1} can be given by

$$T_{d1} = T_{dd}^{clr1} T_{dd}^{cld} T_{dd}^{clr2} \quad (S3a)$$

where T_{dd}^{clr1} is the transmittance of the direct radiation in the atmosphere above the cloud, T_{dd}^{cld} is the transmittance of the direct radiation related to cloud scattering, and T_{dd}^{clr2} is the transmittance of the direct radiation in the atmosphere under the cloud. In this study, we calculate direct radiation

in a diminutive solid angle corresponding to the circumsolar region. Thus, T_{d1} can be approximated as

$$T_{d1} = T_{dd}^{clr} T_{dd}^{cld} \quad (S3b)$$

T_{dd}^{cld} is precomputed for possible cloud conditions and solar incident directions by considering the scattered radiation within the circumsolar region. The details of the computation of T_{dd}^{cld} are specified in the Results.

T_{d2} can be given by integrating solar radiances over the circumsolar region

$$T_{d2} = \frac{1}{\mu_0 F_0} \iint_{\Omega(\theta_0)} I_2 cos\theta d\Omega(\theta_0) \quad (S4a)$$

where $\Omega(\theta_0)$ is the solid angle corresponding to the circumsolar region, θ_0 is the solar zenith angle, and θ is zenith angle. I_2 is the radiance of the downwelling solar radiation related to the multiple reflection between the cloud and land surface, and thus it can be given by assuming an isotropic surface reflection:

$$I_2 = \frac{1}{\pi}(F_{total} - F_1) \quad (S4b)$$

where F_{total} and F_1 are the total downwelling irradiance, i.e., GHI, and the first-order downwelling irradiance, respectively, that can be computed by FARMS. Because θ within the circumsolar region is very close to θ_0, T_{d2} can be approximated as

$$T_{d2} = \frac{I_2 \Omega(\theta_0)}{F_0} \quad (S5)$$

The $\Omega(\theta_0)$ in Eq. (S5) is computed by analyzing the angle between the solar incident and outgoing directions, given as

$$cos\Theta = \mu_0 \mu + (1 - \mu_0^2)^{\frac{1}{2}} (1 - \mu^2)^{\frac{1}{2}} cos\,\varphi \quad (S6)$$

where μ is the cosine value of zenith angle, and φ is azimuth angle. For an individual solar zenith angle, Θ is computed for 50,000 zenith angles and 9,000 azimuth angles in the whole hemisphere ranging from 0° to 90° and 0° to 360°, respectively. The corresponding solid angle related to the circumsolar region is then given by integrating the differential solid angles when $\Theta < \alpha/2$:

$$\Omega(\theta_0) = \iint_{\Theta<\alpha/2} sin\theta d\theta d\varphi \qquad (S7)$$

where α represents the opening angle of the surface-based pyrheliometer. According to the recommendation by the World Meteorological Organization (WMO), a 5° opening angle is assumed for the pyrheliometer on the land surface.

Figure S1 shows the computed solid angle corresponding to the circumsolar region as a function of solar zenith angle. It is seen that the solid angle of the circumsolar region smoothly decreases with the increase in solar zenith angle because of the less condensed differential solid angles in the large solar zenith angles. As a result, the multiple reflection along the direct beam becomes less important with the increase in the solar zenith angle.

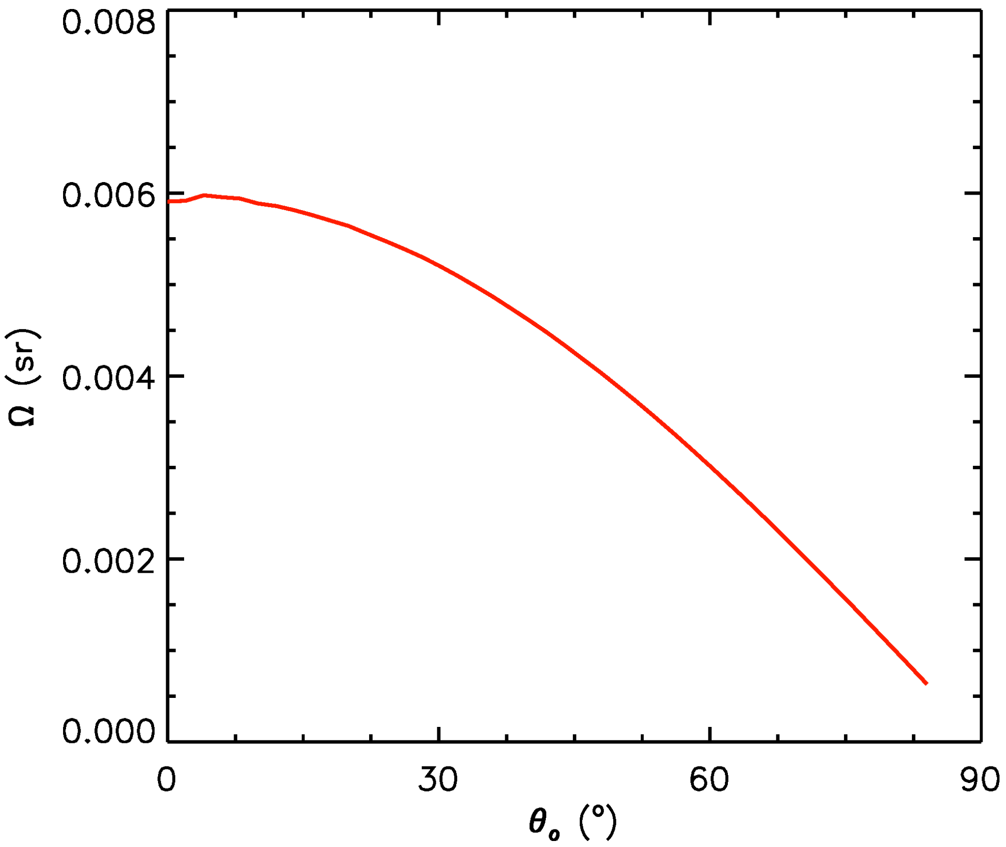

Fig. S1 The solid angle corresponding to the circumsolar region as a function of solar zenith angle. Related to Eq.(1).
